# Strangulated Giant Paraesophageal Hernia Resulting in Ischemia and Perforation: A Case Report

**DOI:** 10.7759/cureus.107393

**Published:** 2026-04-20

**Authors:** Youssef Bahkani, Adelard I De Backer, Koenraad Nieboer, Johan De Mey

**Affiliations:** 1 Department of Radiology, Universitair Ziekenhuis Brussel (UZ Brussel), Brussels, BEL

**Keywords:** case report, gastric ischemia, gastric perforation, paraesophageal hernia, strangulated hernia

## Abstract

An 82-year-old female presented with subacute onset of progressive epigastric pain, persistent retching, and respiratory distress due to a large strangulated paraesophageal hernia complicated by necrosis with perforation. While paraesophageal hernias are often long-standing and asymptomatic, they may evolve insidiously and present with life-threatening complications, e.g., strangulation.

Computed tomography demonstrated marked intrathoracic herniation of the stomach filled with fluid, focal absence of mural enhancement, pneumatosis of the gastric wall, and scattered air densities within a high-attenuation pleural collection (27 HU). Imaging findings were highly suggestive of vascular compromise of the gastric wall with ischemia and perforation. Urgent surgery confirmed gastric wall discoloration consistent with ischemia and necrosis, along with hemorrhagic intrathoracic fluid.

The patient was treated by a distal esophageal resection and partial gastrectomy, followed by reconstruction. This case highlights the importance of recognizing subacute presentation of complicated paraesophageal hernias, as delayed progression may lead to vascular compromise, necrosis, and ultimately perforation. The combination of absent gastric wall enhancement, intrathoracic free air, and high-density pleural fluid due to hemorrhagic content represents critical imaging features that should result in immediate surgical intervention.

## Introduction

Paraesophageal hiatal hernias (PEH) represent a relatively small subset of hiatal hernias but are associated with significant morbidity due to their potential for acute complications. Unlike sliding hiatal hernias, PEH involve herniation of the stomach or other abdominal contents alongside the esophagus, with variable displacement of the gastroesophageal junction depending on the subtype. They are commonly classified into Types II-IV, with Type III representing a mixed form involving both the gastroesophageal junction and fundus. Although often asymptomatic, PEH may present with non-specific gastrointestinal symptoms, such as epigastric discomfort, early satiety, or postprandial fullness, and in advanced cases, with retching, obstruction, or respiratory symptoms due to mass effect. They may also progress to acute emergencies such as gastric outlet obstruction, strangulation, ischemia, and perforation [1]. Prompt diagnosis is essential, as delayed recognition is associated with substantial mortality in complicated cases [2].

## Case presentation

**Figure 1:**
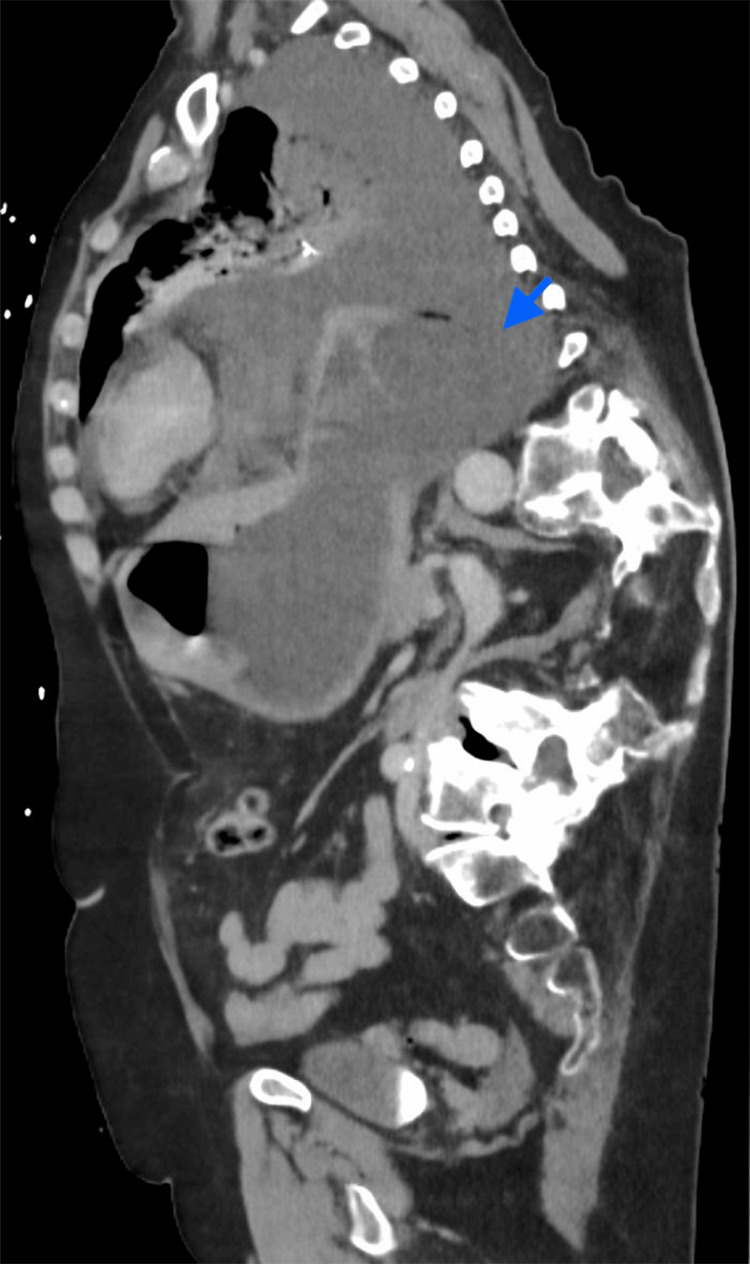
Sagittal contrast-enhanced CT image A large intrathoracic paraesophageal hernia with focal absence of enhancement of the posterior gastric wall (blue arrow), consistent with vascular compromise and transmural ischemia, is seen.

**Figure 2:**
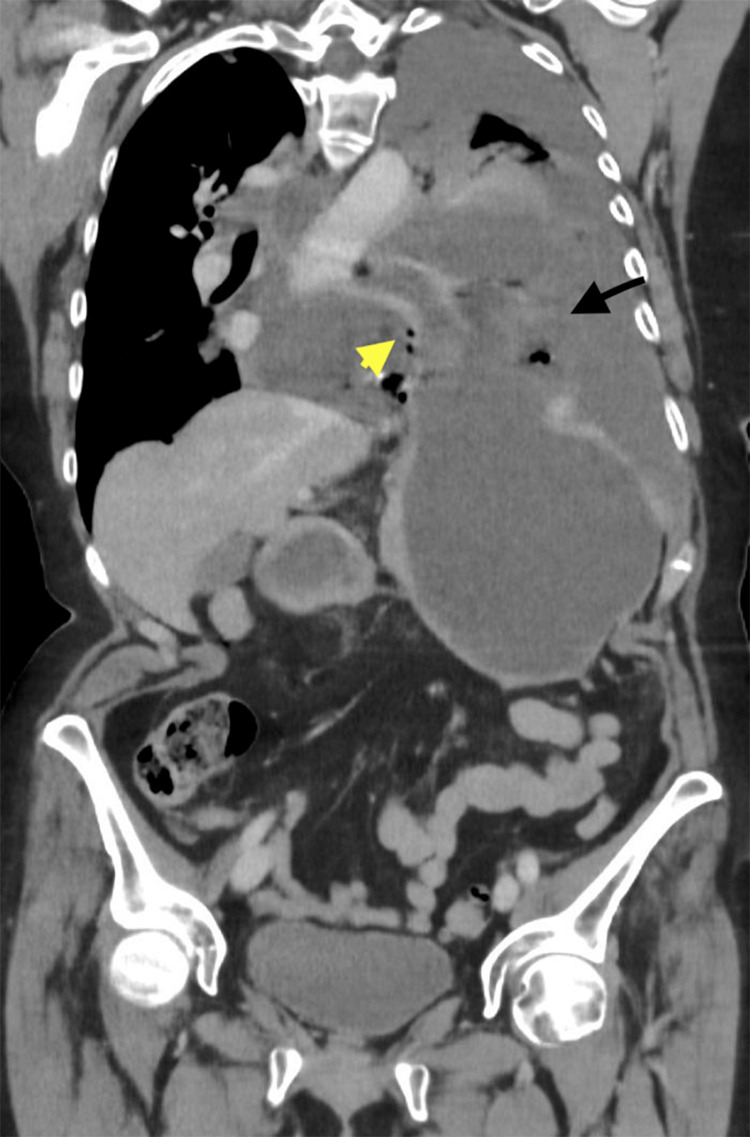
Coronal contrast-enhanced CT image of the thorax A large intrathoracic paraesophageal hernia is seen. A focal absence of gastric wall enhancement (black arrow) is observed, consistent with severe vascular compromise and transmural ischemia. Additionally, free intrathoracic air (yellow arrow) is present, indicating perforation. These findings are highly suggestive of strangulation of the herniated stomach with subsequent necrosis and perforation.

**Figure 3:**
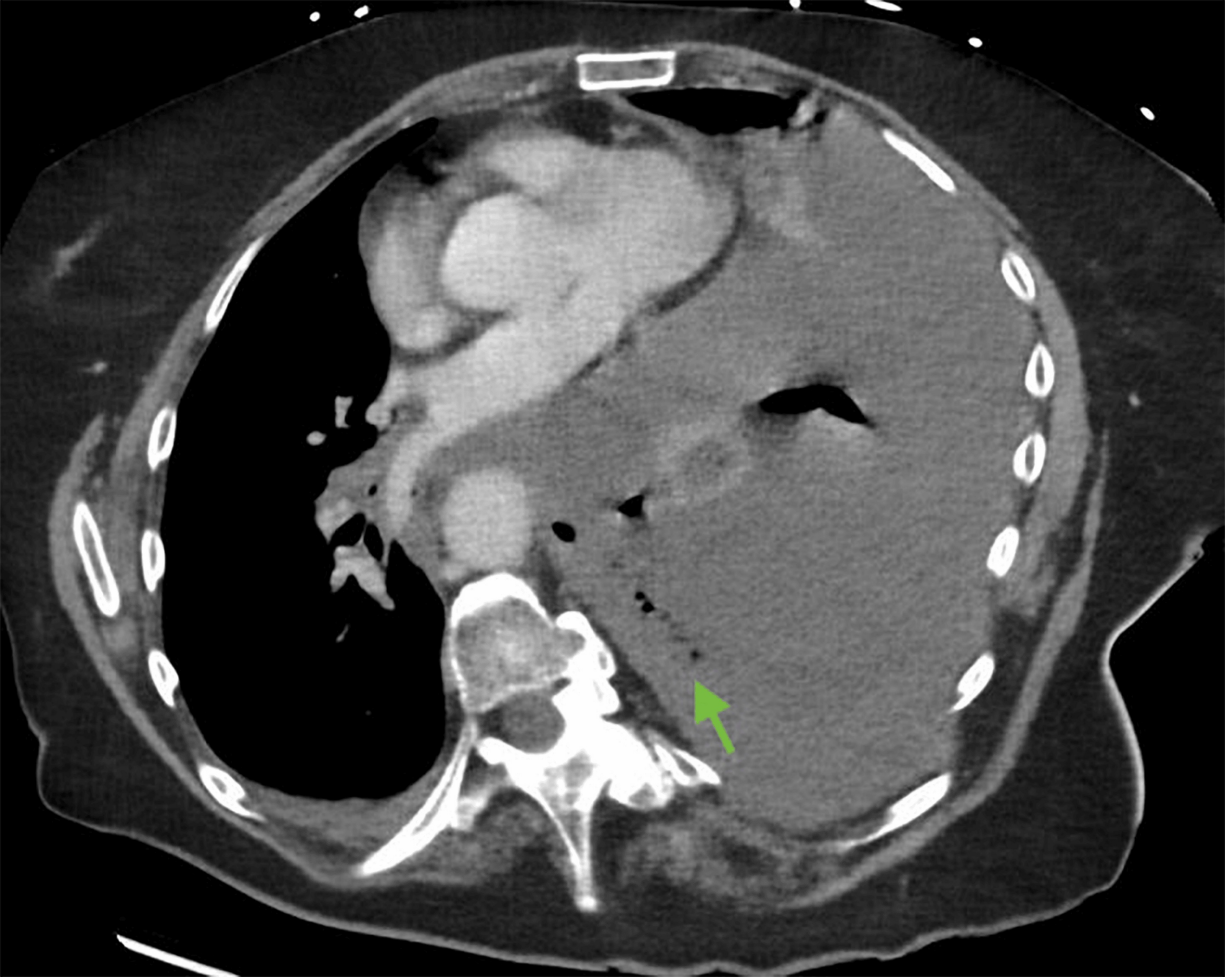
Axial contrast-enhanced CT image Axial contrast-enhanced CT image demonstrating the herniated stomach within the thoracic cavity. Air bubbles are visible within high-attenuation pleural fluid in the left hemithorax (green arrow), suggestive of perforation with associated hemorrhagic effusion.

An 82-year-old female with a history of severe thoracolumbar scoliosis presented with a 3-4 day history of progressive epigastric pain, persistent retching, and respiratory distress. Progressive contrast-enhanced CT of the chest and abdomen demonstrated a large intrathoracic paraesophageal hernia with a dilated, fluid-filled esophagus and stomach. Approximately the entire stomach was herniated into the thoracic cavity with associated mediastinal shift. Focal absence of mural enhancement was noted (Figures 1-2). Additionally, high-attenuation pleural fluid (27 HU) with scattered intrathoracic air densities was present (Figure 3), consistent with hemorrhagic effusion and perforation. These findings were highly suggestive of vascular compromise with gastric ischemia and perforation.

The patient underwent urgent thoracophrenolaparotomy. Intraoperative findings confirmed a Type III paraesophageal hernia with strangulation and extensive transmural ischemic necrosis involving the gastroesophageal junction and proximal stomach. A clear discontinuity of the gastric wall was identified, consistent with perforation. The herniated stomach appeared markedly discolored with areas of non-viable tissue. Additionally, a significant volume of hemorrhagic pleural fluid was encountered, correlating with the increased attenuation observed on CT imaging (Figure 3). These findings confirmed advanced vascular compromise and full-thickness gastric necrosis.

**Figure 4:**
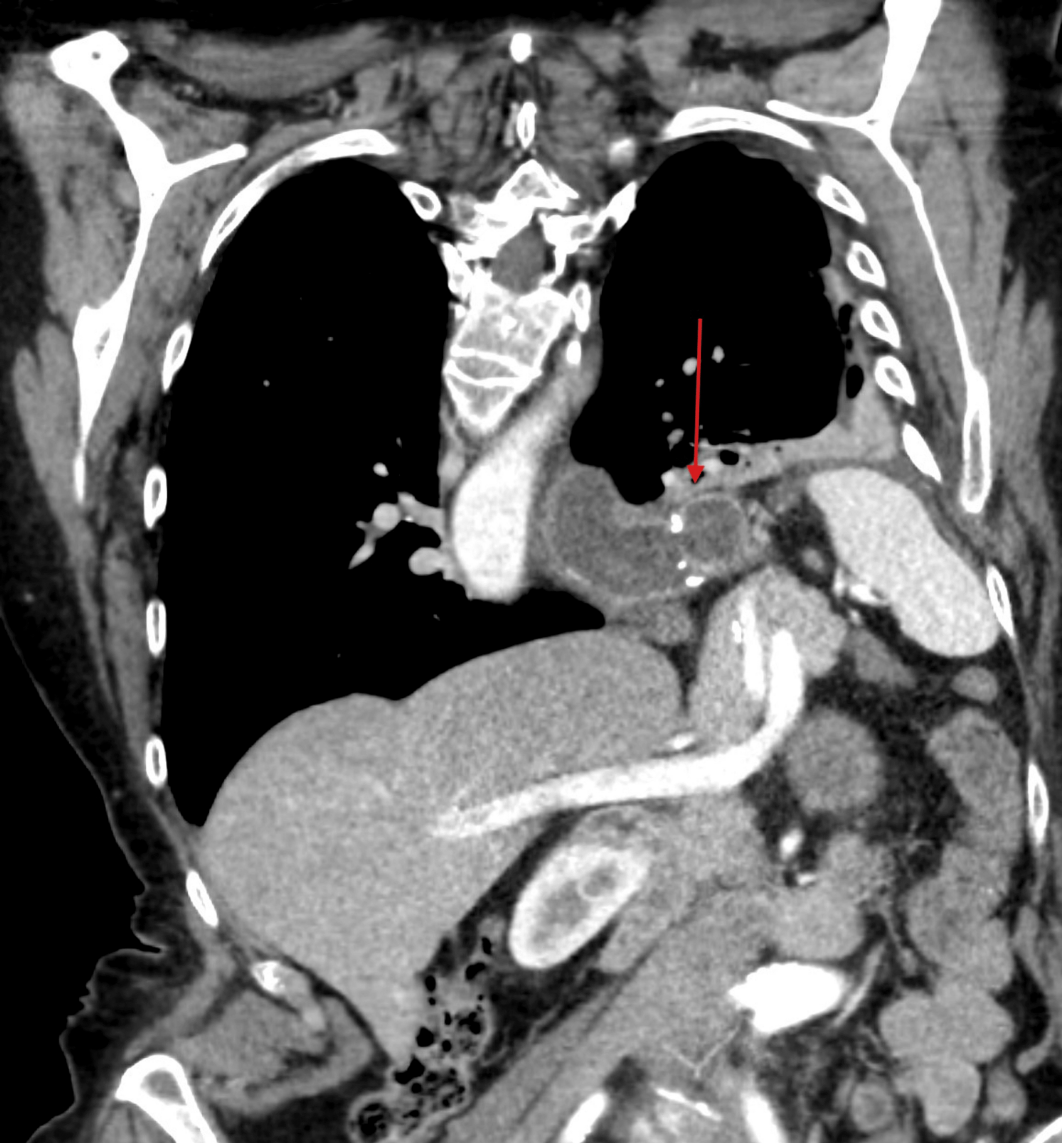
Coronal contrast-enhanced CT image obtained postoperatively demonstrating the intrathoracic esophagogastric anastomosis (red arrow) The anastomotic site appears intact, with no evidence of contrast extravasation, suggesting the absence of leakage.

Distal esophageal resection and partial gastrectomy were performed, followed by reconstruction with intrathoracic esophagogastric anastomosis using a gastric conduit (Figure 4).

Postoperatively, the patient was admitted to the intensive care unit. A follow-up contrast-enhanced CT with oral contrast demonstrated adequate passage through the anastomosis without evidence of leakage or complications. The patient showed gradual clinical improvement with stabilization of respiratory and metabolic parameters.

Intraoperative photographic documentation was not available for this case, as images were not routinely archived in the surgical record. However, operative findings were clearly documented in the surgical report and correlated closely with preoperative imaging findings.

## Discussion

Type III paraesophageal hernias represent a mixed subtype characterized by herniation of both the gastroesophageal junction and a substantial portion of the stomach into the thoracic cavity [3,4]. These hernias are associated with a higher risk of complications compared to sliding hiatal hernias due to mechanical obstruction, impaired gastric emptying, and progressive vascular compromise [5,6].

The pathophysiology of strangulation involves progressive incarceration of the herniated stomach, leading initially to venous congestion, followed by compromised arterial inflow and ultimately transmural ischemia and necrosis [7]. Although the stomach possesses a rich collateral blood supply, extreme distension, torsion, and sustained vascular compromise can overcome these protective mechanisms, resulting in full-thickness necrosis and eventual perforation [7-10]. Gastric necrosis in the setting of paraesophageal hernia is therefore rare and typically reflects advanced disease with a high risk of morbidity and mortality [11-13].

Computed tomography (CT) plays a central role in evaluating suspected complicated paraesophageal hernias. In addition to demonstrating intrathoracic herniation of the stomach and associated mediastinal displacement, CT provides critical information on vascular integrity and complications. Characteristic findings of complicated disease include gastric distension, pneumatosis, focal absence of mural enhancement, and the presence of intrathoracic free air or high-attenuation pleural fluid [8]. Quantitative features, such as the attenuation values of pleural fluid and the extent of herniation, may further support the diagnosis and severity assessment. The combination of pneumatosis and absent mural enhancement is highly suggestive of transmural ischemia and is considered an indication for urgent surgical intervention [8].

Differential considerations may include acute gastric volvulus, perforated viscus from other causes, or complicated sliding hiatal hernia; however, the combination of imaging findings and intrathoracic location strongly supports a strangulated paraesophageal hernia in this case.

In the present case, the increased attenuation of pleural fluid corresponded intraoperatively to hemorrhagic content, while the presence of intrapleural air reflected perforation of the gastric wall. Additional findings, including mediastinal shift, cardiac compression, and pulmonary atelectasis, contributed to the patient’s respiratory compromise and highlight the mass effect that large intrathoracic hernias can exert on adjacent structures [8].

Importantly, this case demonstrates a subacute clinical course over several days, emphasizing that complicated paraesophageal hernias may not always present abruptly but can evolve progressively. Patients may initially present with nonspecific symptoms, such as epigastric pain and retching, before deteriorating due to worsening vascular compromise [6]. This subacute evolution may delay diagnosis and increase the risk of complications if not recognized promptly.

The timing of surgical intervention is a critical determinant of outcome. Elective repair of paraesophageal hernias is associated with significantly lower morbidity and mortality compared to emergent surgery, which is often required in cases of strangulation or perforation [9,11]. Reported mortality rates for complicated paraesophageal hernias remain substantial, particularly in elderly patients with comorbidities [5,9].

Although intraoperative images were not available in this case, the diagnosis was supported by consistent clinical presentation, characteristic radiologic findings, and detailed operative documentation. The strong correlation between CT findings and intraoperative observations reinforces the diagnostic value of imaging in advanced paraesophageal hernia complications.

This case highlights the importance of maintaining a high index of suspicion for complicated paraesophageal hernia in elderly patients presenting with subacute gastrointestinal and respiratory symptoms [14]. Recognition of key CT findings, particularly pneumatosis, absence of mural enhancement, and intrathoracic free air, should prompt urgent surgical management to prevent progression to irreversible ischemia and perforation.

## Conclusions

This case highlights the potential for paraesophageal hiatal hernias to progress insidiously into life-threatening complications such as strangulation, ischemia, and perforation. Recognition of key computed tomography findings, particularly pneumatosis of the gastric wall, absence of mural enhancement, and high-attenuation pleural fluid with intrathoracic air, is critical for early diagnosis. Prompt surgical intervention is essential to prevent severe morbidity and mortality. Clinicians should maintain a high index of suspicion in elderly patients presenting with subacute gastrointestinal and respiratory symptoms, as timely recognition and management significantly improve outcomes.

## References

[1] Dellaportas D, Papaconstantinou I, Nastos C, Karamanolis G, Theodosopoulos T (2018). Large paraesophageal hiatus hernia: is surgery mandatory?. Chirurgia (Bucur).

[2] Kohn GP, Price RR, DeMeester SR (2013). Guidelines for the management of hiatal hernia. Surg Endosc.

[3] Irani F, Mahajan V (2009). Type IV para-oesophageal hiatal hernia: it's a gut feeling. BMJ Case Rep.

[4] Wirsching A, Klevebro F, Boshier PR (2019). Contemporary management of paraesophageal hernia. Br J Surg.

[5] Sihvo EI, Salo JA, Räsänen JV, Rantanen TK (2009). Fatal complications of adult paraesophageal hernia: a population-based study. J Thorac Cardiovasc Surg.

[6] Wee M, Liu DS, Thompson SK (2021). Acute gastric dilatation: a life-threatening early complication following laparoscopic hiatus hernia repair. ANZ J Surg.

[7] Fukai S, Kubota T, Mizokami K (2019). Gastric perforation secondary to an incarcerated paraesophageal hernia. Surg Case Rep.

[8] Millet I, Orliac C, Alili C, Guillon F, Taourel P (2014). Computed tomography findings of acute gastric volvulus. Eur Radiol.

[9] Shea B, Boyan W, Decker J (2019). Emergent repair of paraesophageal hernias and the argument for elective repair. JSLS.

[10] Deliwala SS, Hussain MS, Ponnapalli A, Bachuwa G, Gurvits GE (2021). Black oesophagus, upside-down stomach and Cameron lesions: cascade effects of a large hiatal hernia. BMJ Case Rep.

[11] Luketich JD, Nason KS, Christie NA, Pennathur A, Jobe BA, Landreneau RJ, Schuchert MJ (2010). Outcomes after a decade of laparoscopic giant paraesophageal hernia repair. J Thorac Cardiovasc Surg.

[12] Schuchert MJ, Adusumilli PS, Cook CC (2011). The impact of scoliosis among patients with giant paraesophageal hernia. J Gastrointest Surg.

[13] Longchamp G, Andres A, Abbassi Z (2021). Gastric necrosis following a hiatal hernia: a case report. Int J Surg Case Rep.

[14] Stylopoulos N, Rattner DW (2005). The history of hiatal hernia surgery: from Bowditch to laparoscopy. Ann Surg.

